# Cell Shortening and Calcium Homeostasis Analysis in Adult Cardiomyocytes via a New Software Tool

**DOI:** 10.3390/biomedicines10030640

**Published:** 2022-03-10

**Authors:** Lorenzo Fassina, Maria Rita Assenza, Michele Miragoli, Andrea M. Isidori, Fabio Naro, Federica Barbagallo

**Affiliations:** 1 Department of Electrical, Computer and Biomedical Engineering (DIII), University of Pavia, 27100 Pavia, Italy; lorenzo.fassina@unipv.it; 2 Institute of Biochemistry and Cell Biology, CNR, 00015 Monterotondo, Italy; mariarita.assenza@ibbc.cnr.it; 3 Department of Anatomical, Histological, Forensic and Orthopedic Sciences, Sapienza University, 00161 Rome, Italy; fabio.naro@uniroma1.it; 4 Department of Medicine and Surgery, University of Parma, 43126 Parma, Italy; michele.miragoli@unipr.it; 5 Humanitas Research Hospital—IRCCS, 20089 Rozzano, Italy; 6 Department of Experimental Medicine, Sapienza University, 00161 Rome, Italy; andrea.isidori@uniroma1.it

**Keywords:** Ca^2+^ transient, fraction shortening, software, adult ventricular cardiomyocytes, β-Adrenergic receptor

## Abstract

Intracellular calcium (Ca^2+^) is the central regulator of heart contractility. Indeed, it couples the electrical signal, which pervades the myocardium, with cardiomyocytes contraction. Moreover, alterations in calcium management are the main factors contributing to the mechanical and electrical dysfunction observed in failing hearts. So, simultaneous analysis of the contractile function and intracellular Ca^2+^ is indispensable to evaluate cardiomyocytes activity. Intracellular Ca^2+^ variations and fraction shortening are commonly studied with fluorescent Ca^2+^ indicator dyes associated with microscopy techniques. However, tracking and dealing with multiple files manually is time-consuming and error-prone and often requires expensive apparatus and software. Here, we announce a new, user-friendly image processing and analysis tool, based on ImageJ-Fiji/MATLAB^®^ software, to evaluate the major cardiomyocyte functional parameters. We succeeded in analyzing fractional cell shortening, Ca^2+^ transient amplitude, and the kinematics/dynamics parameters of mouse isolated adult cardiomyocytes. The proposed method can be applied to evaluate changes in the Ca^2+^ cycle and contractile behavior in genetically or pharmacologically induced disease models, in drug screening and other common applications to assess mammalian cardiomyocyte functions.

## 1. Introduction

Calcium (Ca^2+^) plays a critical role in maintaining the adequate contractile function of the cardiomyocytes [1]. Physiologically, changes of cytoplasmic calcium concentration ([Ca^2+^]_i_) link the plasma membrane depolarization (action potential, AP) to contraction, a process named excitation-contraction (E-C) coupling. The AP spreads between sarcomeres, leading to Ca^2+^ influx (*I*_Ca_) through the voltage-gated L-type Ca^2+^ (LTCC) lining in the T-tubules. Ca^2+^ ions inflow induces Ca^2+^ release from the sarcoplasmic reticulum (SR) through the ryanodine receptors (RyRs) through a process known as “Ca^2+^-induced Ca^2+^-release” (CICR). This mechanism leads to a 10-fold increase in the free intracellular [Ca^2+^]_i_ and provides a sufficient amount of Ca^2+^ to bind troponin-C and promote cardiomyocytes contraction. Force of contraction is dependent on the amount of Ca^2+^ bound to troponin-C and it will be a function of both the magnitude and duration of the rise of [Ca^2+^]_i_. Cardiomyocyte relaxation is induced by rapid Ca^2+^ reuptake in the SR due to the activation of the sarcoplasmic/endoplasmic reticulum Ca^2+^-ATPase2a (SERCA2a) pump. Intracellular Ca^2+^ signals then include transients, oscillations, and propagating waves [2,3,4,5]. Spatial and temporal control of [Ca^2+^]_i_ is finely tuned, so it is not surprising that its abnormalities are associated with cardiac dysfunctions [6]. Indeed, heart failure is characterized by misregulation of Ca^2+^ homeostasis [7,8], which leads to cardiomyocytes shortening impairment [9,10]; as a consequence, the investigation of Ca^2+^ cycling and the correlated contractile function in isolated cardiomyocytes, widely used as an in vitro model, provides important information regarding their health status. Moreover, the analysis of E-C coupling is used to study the effects of different drugs with potential inotropic or lusitropic effects to confirm their effectiveness [11,12,13].

Several methods have been applied to monitor cardiomyocyte shortening and/or Ca^2+^ transients. Regarding the analysis of contractile function, among the simplest is edge detection in a video recording. This method allows the detection of cardiomyocytes’ dimension variation and post-analysis of the video via an algorithm. The implementation of computational detection methods is reaching the ability to monitor cells with different shapes and contraction axis and to determine their fraction shortening, not only in isolated adult cardiomyocytes, which show a well-determined contraction [14,15], but also in iPSCs-derived cardiomyocytes [16,17] and in neonatal cardiomyocytes [18].

[Ca^2+^]_i_ has been monitored by plate-based assays, spectrofluorimetry, and flow cytometry, but usually, fluorescence microscopy and Ca^2+^ indicator dyes (such as Fluo-4 AM and Fura-2) are used due to their feasibility and low cost. After time-lapse video acquisition, the temporal Ca^2+^ signal is evaluated as time-dependent, normalized, mean-in-space fluorescence (f/f_0_) within a user-defined area or region of interest (ROI); the subsequent analysis is considerably time consuming.

Despite the existence of some computational solutions for the analysis of cardiomyocyte fraction shortening and Ca^2+^ transients, the use of them requires good knowledge of programming. Some software, such as Video Sarcomere Length (VSL) (901D, Aurora Scientific, Aurora, ON, Canada), has a user-friendly interface; however, VSL performs the shortening detection via two length calculation methods, using the fast Fourier transform analysis that requires high-resolution videos, high image magnification, and high-speed camera [18]. Other commercial software, such as IonOptix system (IonOptix, Westwood, MA, USA), presents different modules for both shortening detection and Ca^2+^ signal analysis, but is not cost effective. Thus, the laboratories are often unable to afford the substantial costs involved in purchasing both commercial software and video acquisition equipment.

We aimed to develop a simple tool that allows the researchers with basic programming skill to measure the contractile parameters of primarily isolated adult cardiomyocytes.

Therefore, this work aims to describe a new analysis tool that can be used on videos or image stacks loaded in ImageJ-Fiji (https://fiji.sc/, accessed on 7 March 2022) and then analyzed by our custom-made MATLAB^®^ scripts in order to evaluate cell fraction shortening and Ca^2+^ transient and kinematics/dynamics of primary isolated adult cardiomyocytes (MATLAB^®^, Release R2020a, The MathWorks, Inc., Natick, MA, USA). These MATLAB^®^ scripts can perform Ca^2+^ transients and shortening analysis of cells that are not necessarily positioned horizontally. Our method only requires recorded videos, even at low resolution, captured in wide-field fluorescence and bright-field microscopy, without the requirement of additional equipment. In addition, our custom-made scripts, when compiled and given upon request, can be easily run without a license since MATLAB^®^ software house provides, for free, the runtime which enables the execution of scripts on computers without an installed version of MATLAB^®^ (https://it.mathworks.com/help/compiler/install-the-matlab-runtime.html, accessed on 7 March 2022).

## 2. Materials and Methods

### 2.1. Mouse Husbandry

All procedures involving mice were completed following the policy of the Italian National Institute of Health (Protocol no. 145/2017-PR) and with the ethical guidelines for animal care of the European Community Council (Directive 86/609/ECC). Male C57BL/6N mice (2–4 months old) were obtained from the Charles River Laboratories Italia (Calco, Italy) and were housed in the DAHFMO, Sapienza University of Rome, under 12 h light/dark cycles, at a constant temperature, with food and water ad libitum.

### 2.2. Isolation of Primary Adult Cardiomyocytes

Adult ventricular myocytes (AVMs) were isolated as previously described [9]. Briefly, mice were injected with 5000 units/kg heparin, sacrificed by cervical dislocation, and hearts were quickly removed and placed in cannulation buffer (NaCl 120 mM, KCl 5.4 mM, NaH_2_PO_4_ 1.2 mM, NaHCO_3_ 20 mM, MgSO_4_ 1.2 mM, glucose 5.6 mM, 2,3-Butanedione monoxime (BDM) 10 mM, taurine 20 mM, pH = 7.33). The heart was gently cannulated via the aorta in a Langendorff perfusion apparatus and filled with cannulation buffer. The hearts were perfused initially with a predigestion buffer (0.05% collagenase type II (Worthington Biochemical, Lakewood, NJ, USA) and 0.01% protease type XIV (Sigma-Aldrich, MO, USA) solution with no calcium) and then with digestion buffer (0.2% collagenase type II, 0.04% protease type XIV, and 50 μM CaCl_2_) for complete digestion. The heart was cut below the atria into a Petri dish containing 5 mL of digestion buffer when the heart became soft. The minced heart was then transferred into a stopping buffer (12.5 μM CaCl_2_ and 10% FBS). The isolated myocytes were recovered with a gradient of calcium cannulation buffer to 1 mM CaCl_2_. Freshly isolated myocytes were washed three times and suspended with beating buffer (NaCl 120 mM, KCl 5.4 mM, NaH_2_PO_4_ 1.2 mM, MgSO_4_ 1.2 mM, HEPES 20 mM, glucose 5.5 mM, CaCl_2_ 1 mM, pH = 7.1, filtered) to remove BDM before shortening and calcium transient assay.

### 2.3. Calcium Transient and Contraction

Freshly isolated AVMs were loaded with Fluo-4 AM (5 μM; Molecular Probes, Grand Island, NY, USA) for 15 min. Cells were then placed in the middle of a glass-bottomed dish with 3 mL beating buffer and settled for 5 min. Platinum electrodes were placed near the cell and paced the cell at 1 Hz (with a voltage of 50 V) using the SD9 stimulator (Grass Technology, Warwick, RI, USA) as previously described [10]. The calcium transient and contractile events of cardiomyocytes were recorded via an inverted microscope (Zeiss AX10, Dublin, CA, USA) at 20× magnification, enabling the observation of 5–10 rod-shaped cardiomyocytes with clear striations per field of view (using the MetaMorph software, Molecular Devices, Sunnyvale, CA, USA). The following settings were applied: 200 frames per movie, exposure time 25 ms, movie length 5 s. Isoproterenol 100 nM was added into the Petri dish after the first movie (control) was acquired. The calcium transients were recorded with excitation at 488 nm and emission collected at >505 nm.

### 2.4. Statistical Analysis

All data were expressed as mean ± SEM. Statistical analysis was performed using GraphPad Prism 6 software (La Jolla, CA, USA). The sample size for each group is shown in the figure legends. The cells came from at least three sets of independent experiments. Differences between two groups (untreated; isoproterenol—ISO) were evaluated by Welch’s t-test. The relationships between τ of decay, τ to peak, fraction shortening, and Ca^2+^ transient amplitude were compared between groups by linear regression analysis (confidence interval: 95%) with Spearman correlation coefficient. *p* < 0.05 was defined as statistically significant.

## 3. Results

### 3.1. Analysis of Cell Shortening and Kinematics/Dynamics of Primary Isolated Adult Cardiomyocytes

**Scheme 1:**
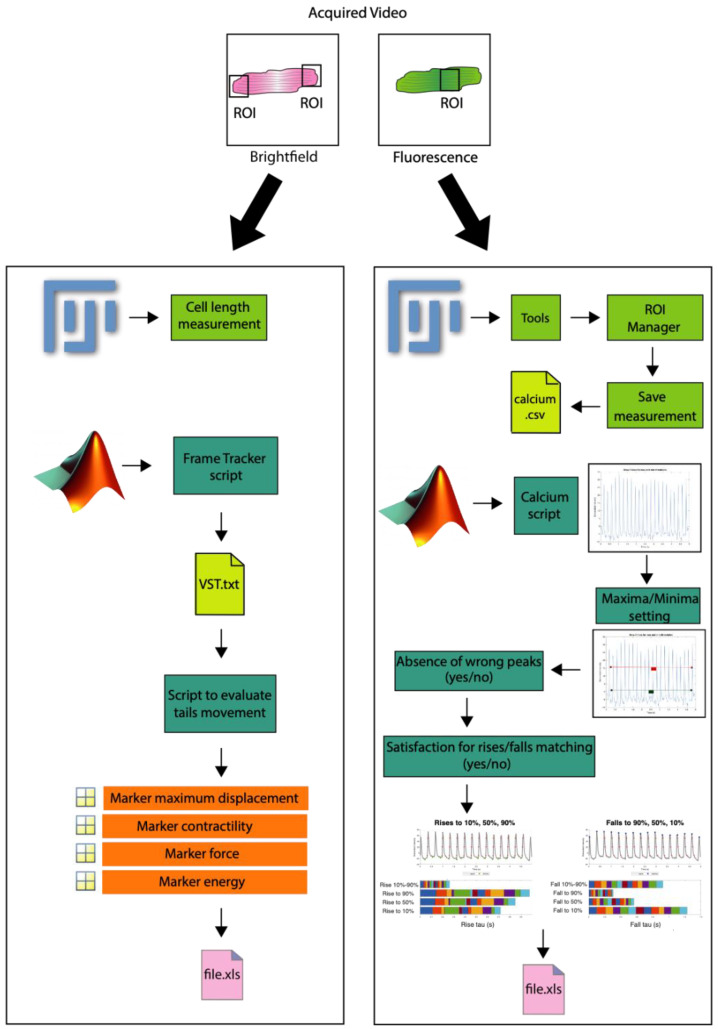
Overview of ImageJ-Fiji/MATLAB^®^ workflow. Green blocks indicate basic steps on Fiji software. Cerulean blocks show MATLAB^®^ steps (i.e., script execution with user’s choices during analysis). Orange blocks indicate results. Pink blocks are related to the saving of results. On the left and right panels, analyses for contraction shortening and calcium transients are represented, respectively. Two in-between result files are generated: (1) VST.txt contains the tails’ tracking, the input for further analysis with MATLAB^®^; (2) calcium.csv has the calcium data generated by Fiji [19], the input for further analysis with MATLAB^®^.

To comprehensively study the contractile cycle of adult ventricular myocytes (AVMs), a new simple analysis tool was developed based on ImageJ-Fiji/MATLAB^®^ software in order to evaluate fraction shortening (Scheme 1).

**Figure 1:**
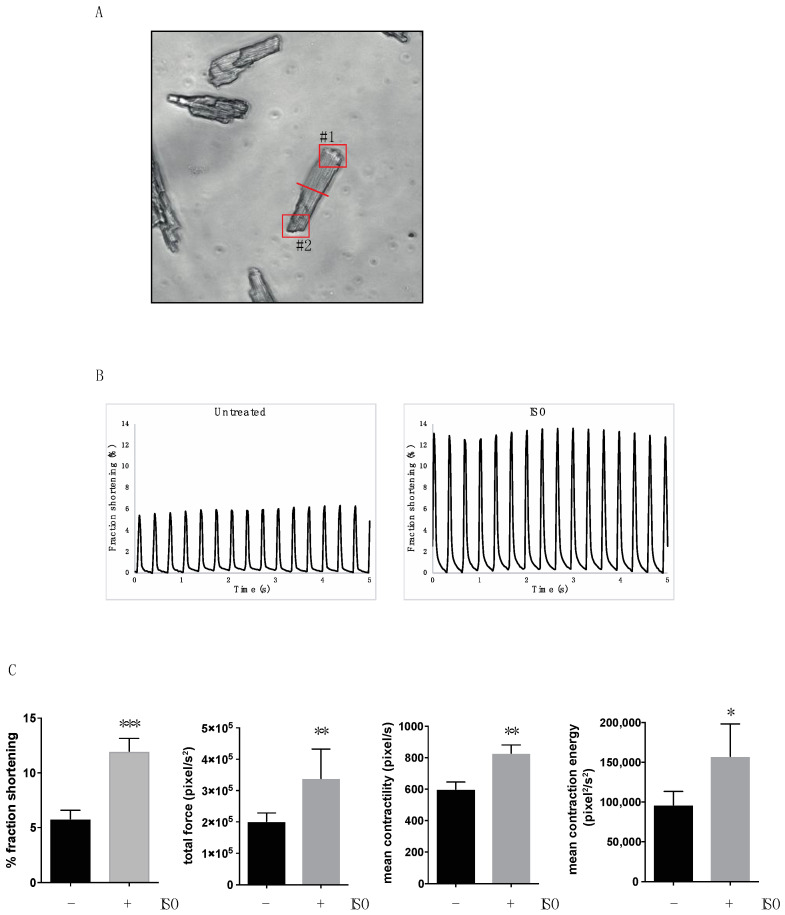
(**A**) Representative image of adult ventricular murine cardiomyocytes that undergo further analysis. The red line indicates the mid cell region. The cell tails are numbered. (**B**) Fraction shortening (%) of untreated cell (left panel) and an isoproterenol-stimulated cell (right panel). (**C**) Statistics for cell shortening and cell kinematics/dynamics. Results for fraction shortening, total force, mean contractility, and mean contraction energy at basal (untreated) and after isoproterenol stimulation (100 nM), obtained from three independent experiments (total cell number equal to 60), are reported as mean ± SEM in the histograms bar chart, * *p* < 0.05, ** *p* < 0.01, *** *p* < 0.001.

AVMs, once electrically paced at 1 Hz, shorten by moving both tails, whereas their central region remains fixed (Figure 1A). According to this principle, the uncontracted length L_0_ (pixel) of each cardiomyocyte was measured by ImageJ-Fiji (https://fiji.sc/, accessed on 7 March 2022). The movement of each cell tail was evaluated as previously described [20,21,22,23]. In this manner, it was possible to measure the mean maximum displacement (pixel) of both tails: MD_1_ and MD_2_, respectively. Subsequently, the cell fraction shortening (%) was calculated as (MD_1_ + MD_2_)/(L_0_/2) (Figure 1B).

Similarly, the total contraction force (pixel/s^2^) was calculated as the sum of the tails’ force (each measured as previously reported [20,21,22,23,24]), whereas the mean contractility (pixel/s) and the mean contraction energy (pixel^2^/s^2^) were calculated as the average of the tails’ parameters (each measured as previously reported [20,21,22,23,24]).

β-Adrenergic (β-AR) stimulation was used to obtain a positive lusitropic and inotropic effect to check the validity of our method. As reported in Figure 1B,C, as expected, AVMs treated with isoproterenol (a non-selective β-AR agonist) displayed an increase of tails’ mean maximum displacement resulting in an elevation of fraction shortening (Figure 1C). The averages of basal-untreated (5%) and isoproterenol-stimulated (12%) fraction shortening were in line with the results obtained via alternative methods [9,25,26], demonstrating the reliability of our analysis tool. Moreover, in the isoproterenol stimulation, the enhanced fraction shortening parallels the augmentation of total contraction force, mean contractility, and mean contraction energy (Figure 1C). All the data obtained indicate that the developed scripts give a reproducible determination of contraction parameters in AVMs.

### 3.2. Analysis of Ca^2+^ Transient in Primarily Isolated Adult Cardiomyocytes

The right panel in Scheme 1 depicts all the steps necessary to analyze Ca^2+^ transients of Fluo-4-AM-loaded cardiomyocytes starting from acquired image sequences.

**Figure 2:**
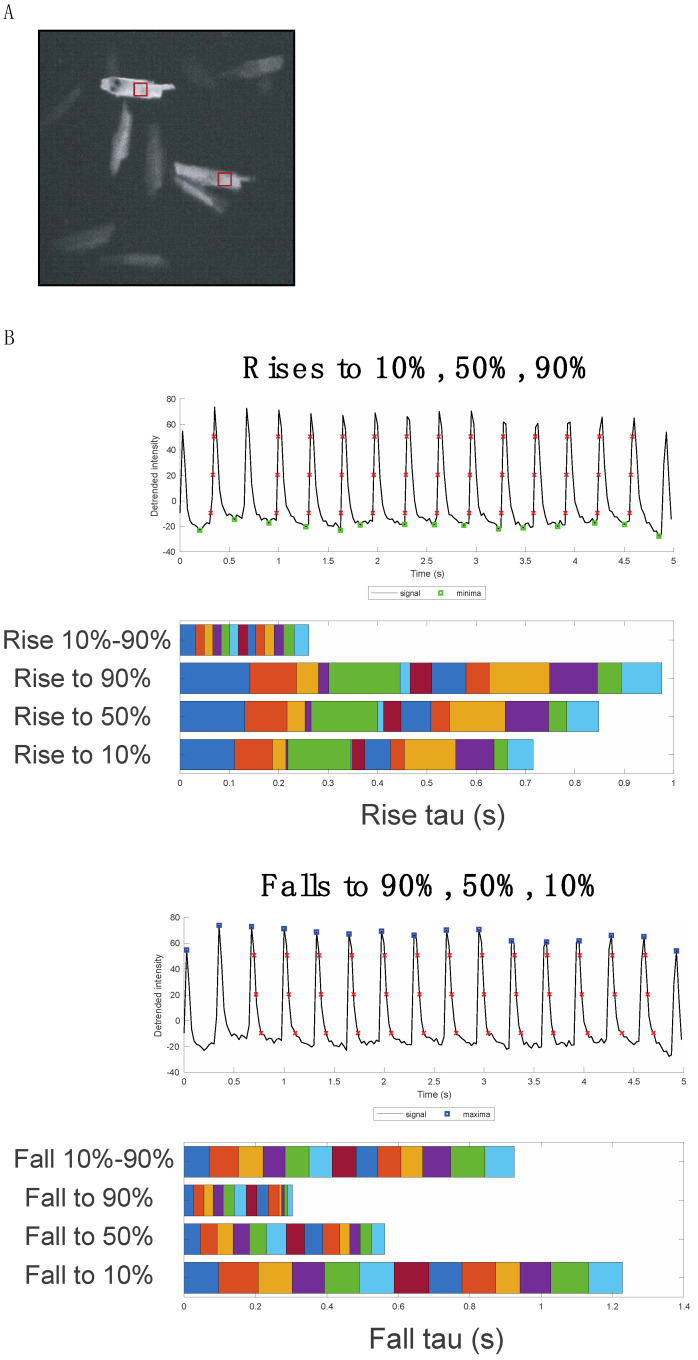
(**A**) Representative image of adult cardiomyocytes for the analysis of Ca^2+^ transients via ImageJ-Fiji. (**B**) The detrended intensity of the normalized mean-in-space fluorescence (f/f_0_) for an untreated cell within a user-defined ROI (red box in panel **A**) is shown. Each Ca^2+^ transient is calculated from resting/baseline to peaks. The red crosses are placed where the fluorescence signal is at its 10%, 50%, and 90% for both rise and fall patterns (upper and lower panel, respectively). Some transients have no crosses since they are considered outliers, whereas the last transients are not measured, given their incomplete transitions. In general, there are reported, in the horizontal stacked histogram bars, the τ related to 10%, 50%, 90%, and 10–90% range for both rise and fall patterns and for each detected transition (each transition is depicted with a specific color).

According to http://akiramuto.net/archives/148 (accessed on 7 March 2022) and [19], initiating from the first frame in ImageJ-Fiji software, the intensity of the normalized mean-in-space fluorescence (f/f_0_) was measured within a user-defined ROI (Figure 2A), in particular, via background subtraction in each frame of Ca^2+^ analysis.

It is good practice, in signal analysis, to detrend the signal vs. time to remove the *y*-axis shifts of the signal baseline during the observation time; for this reason, via a custom-made script in MATLAB^®^ (Release R2020a, The MathWorks, Inc., Natick, MA, USA), we have detrended the fluorescence signal, obtaining the typical pattern of the Ca^2+^ transients (Figure 2B).

As shown in Figure 2B, this pattern is a bi-level waveform transition, where the low level is the baseline and the high level is the peak fluorescence. For the bi-level waveform transitions, the IEEE Standards Association published a standard entitled “*181-2011—IEEE Standard for Transitions, Pulses, and Related Waveforms*” (https://standards.ieee.org/standard/181-2011.html, accessed on 7 March 2022), which is the state of the art for that kind of signal analysis. In particular, in MATLAB^®^, there is an implementation of the preceding IEEE standard (functions “risetime” and “falltime” of Signal Processing Toolbox™, The MathWorks, Inc., Natick, MA, USA): on the statistical basis between the various transitions from peaks to baseline, the red crosses are placed where the fluorescence signal is correctly recognized at its 10%, 50%, and 90% for both rise and fall patterns (Figure 2B, upper and lower panel, respectively). Specifically, we are interested in measuring, for each transient, a time span called “the transient’s τ”, which is a measure of the “lifetime” of the transient: we redefine τ as the time to fall from a fluorescence peak to 10% of the fluorescence signal (we can also elect a different percentage; see below). As we can observe, some transients have no red crosses. They are considered outliers on a statistical basis, whereas the last transients are not measured because they are incomplete transitions. In the horizontal histograms, the τ related to 10%, 50%, 90%, and 10–90% is reported in stacked style to appreciate the beat-to-beat variability, for both rise and fall patterns (Figure 2B). All the obtained plots can be saved and customized for publications.

**Figure 3:**
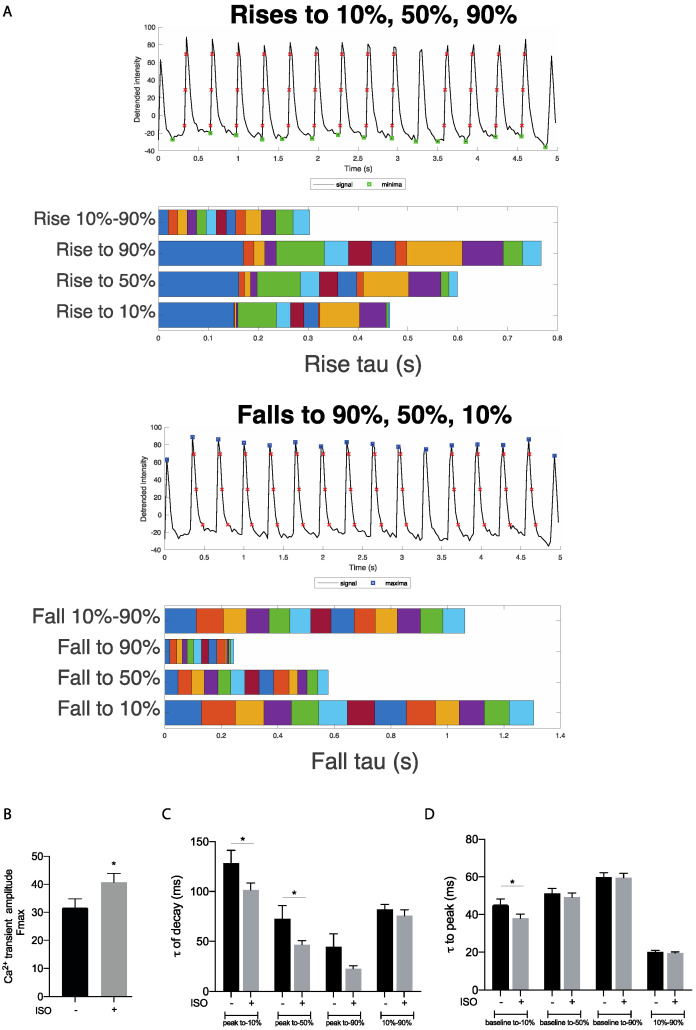
(**A**) Detrended intensity of the normalized mean-in-space fluorescence (f/f_0_) for an isoproterenol-stimulated cell within a user-defined ROI. The red crosses are placed where the fluorescence signal is at its 10%, 50%, and 90% for both rise and fall patterns (upper and lower panel, respectively). (**B**–**D**) Results for Ca^2+^ transient’s amplitude F_max_, τ-of-decay (ms), and τ-to-peak (ms) at basal (untreated) and after isoproterenol stimulation (100 nM), obtained from three independent experiments (total cell number equal to 60), are reported as mean ± SEM in the histograms bar chart, * *p* < 0.05.

Moreover, by averaging in time, we calculated the Ca^2+^ transient amplitude F_max_ of the fluorescence as the mean span from peaks to baseline (Figure 3B). As for fraction shortening analysis, β-AR stimulation was used in order to obtain a positive lusitropic and inotropic effect to verify the validity of our method. Isoproterenol-treated cells displayed a significant increase of Ca^2+^ transient amplitude, a significant reduction of the time for the transient to reach 10% during the rising phase (Figure 3D), and a return to the 10% or to the 50% of the peak during the recovery phase of the transient (Figure 3C), indicating a faster Ca^2^^+^ reuptake as expected after β-ARs activation (Figure 3A–C). No statistically significant differences were observed for the τ to peak from baseline to 50% or to 90% (Figure 3D). When τ-to-peak (τ related to 10%, 50%, and 90%) recorded from individual isoproterenol-treated cells against untreated cells was plotted, the correlation coefficient (r) estimated from the linear fitting displayed a negative correlation (Supplementary Figure S1A–C). An analogous negative correlation was found when τ-to-fall, in the same condition, was analyzed (Supplementary Figure S1D–F). Additionally, the durations of the τ-of-decay and τ-to-fall were directly correlated when β-ARs were activated, which suggests that cardiomyocytes, which show a shorter time to peak, also have a shorter time of decay (Supplementary Figure S1G–I). Finally, a positive correlation was found when Ca^2+^ transient amplitude was compared to fraction shortening and a faster τ-of-decay or τ-to-fall corresponds to a more efficient shortening (Supplementary Figure S2A–C).

## 4. Discussion

In the present study, we developed and verified (via “reobtaining” well-known knowledge about β-Adrenergic stimulation) a combined ImageJ-Fiji/MATLAB^®^ approach that enables systematic analysis of the fundamental calcium transient’s parameters and the related shortening/recovery cycle in mouse adult cardiomyocytes, also observing many cells per image.

An easy-to-use method to quantify cardiomyocyte shortening and Ca^2+^ cycle is fundamental to determine the physiological consequence of various genetic manipulations and to identify potential therapeutic targets [27].

Some platforms are available to quantify heart or cardiomyocytes shortening or Ca^2+^ transients, but often they are not user-friendly, needing specific instrumentation and thus becoming expensive, such as Video Sarcomere Length (VSL) (Aurora Scientific, Canada) [18] or other commercial software, such as the IonOptix system (IonOptix, Westwood, MA, USA) [28]. In particular, in comparison to IonOptix technology, our methodology is already applicable for any existing inverted or upright microscope. Moreover, we can perform analysis, without the need of sarcomeres’ horizontal reorientation, on each single beat separately from the other beats and, especially, for each single cell of the experiment. In addition, because sarcomere regularity is not a requirement, we can investigate not only adult, but also cardiac cell lines (e.g., HL1 cells), and primary neonatal and iPSC-derived cardiomyocytes. In general, with our method, only quick training is requested for the user, and, from a practical point of view, we increased the measurement speed, studying almost all the cardiomyocytes in a single preparation included in the field of view, which becomes the main advantage when they are isolated from a costly source, such as rare transgenic mice and human biopsy. Finally, and most importantly, our technology provides kinematic information of the cells such as force and energy; high-throughput functional kinematic investigation of E-C coupling in isolated cardiomyocytes has been attempted, but again it needs dedicated and expensive hardware/software. Other platforms, despite their easy usability, only allow measuring cardiomyocytes shortening, without considering Ca^2+^ handling [14,29].

Here, we developed and tested straightforward MATLAB^®^ scripts, reducing the time consumed in the analysis and providing a comprehensive set of functional parameters from isolated adult cardiomyocytes. Given the fact that the custom scripts can be used for the analysis of videos and time-lapse image sequences, it is natural to consider their use for moving cells (Scheme 1, left) and/or for spiking cells (Scheme 1, right): in moving cells, for example, it is possible to track the contractile response following exposure to different drugs given in sequence over time. A rapid Ca^2+^ cycle phenotyping of spiking cells, such as induced pluripotent stem cells-derived cardiomyocytes (iPSC-CM), stained with calcium dye or infected with GFP- or RFP-based calcium probes, would be possible. Quantitative and qualitative assessment of Ca^2+^ transient parameters can be performed in pathological conditions, e.g., in patient-specific cell line carrying arrhythmogenic diseases such as arrhythmogenic right ventricular dysplasia (ARVD) and catecholaminergic polymorphic ventricular tachycardia (CPVT) [30].

Even if alternative inexpensive methods for cardiac contraction have been proposed [29], the main advantage of our Ca^2+^ transient analysis is the implicit statistics to identify the pattern of the bi-level (peak-baseline) waveform transitions, discarding the outliers. This signal analysis method is defined in an IEEE standard and represents the state of the art for such signals, it is easy to be implemented in customized scripts and, specifically, there is no need to hypothesize mathematical models of the transient temporal pattern, thus avoiding data fitting and fitting interpretation. In other words, the main information for the transient’s τ is easily obtainable without using mathematical models (e.g., as performed in [31]).

The reliability of our tool was verified using isolated adult murine cardiomyocytes, giving expected results (Figure 1, Figure 2 and Figure 3) when a known β-Adrenergic input was given. More specifically, our script was able to detect a significant increase of Ca^2+^ transient amplitude and, also, a decrease of the transient time throughout the recovery phase, as expected after β-ARs activation. Moreover, the temporal patterns of the Ca^2+^ transient and Ca^2+^ transient amplitude, at basal (untreated) and after isoproterenol stimulation, obtained with our software tool, were identical to those showed by CalTrack, an alternative validated software [32].

It is worth noting that isoproterenol stimulation induces biphasic dose-dependent cAMP and PKA activation in cardiomyocytes: at picomolar doses, a transient increase in the initial peak response, and, at nanomolar doses, a saturated and sustained increase has been observed. This dose-dependent response, which dictates PKA substrate-specificity and cardiac contraction response, could be investigated by analyzing fractional cell shortening, Ca^2+^ transient amplitude, and the kinematics/dynamics parameters with our software tool, providing a real-data-based novel insight into cardiovascular physiology.

We have highlighted the beat-to-beat variability emerging from the transient’s analysis (Figure 2 and Figure 3): whereas commercial tools make a merger of the recorded beats before evaluating specific parameters, here, on the contrary, we can separate the measures emerging from each beat for a more suitable statistics. In other words, the statistics could be performed for the entire set of recorded beats, or, if necessary, for subsets of them (for example, it could be of interest to conduct the temporal study of arrhythmias or to study parameters under the effect of drugs during the recording time).

We are aware that, to date, the gold standard for such measurement in adult cardiomyocytes is a technology, namely IonOptix [33]. Here we showed, using free ImageJ-Fiji in the pipeline with our custom scripts, the capability to select more than one ROI independently from sarcomere regularity and organization. Indeed, the algorithm [21,23] provides data about cell kinematics without keeping in consideration sarcomere shortening and can be easily applicable to cells that displayed non-uniform sarcomere organization such as neonatal cardiomyocytes or iPSC-CM [34].

One limitation is that our temporal resolution (i.e., 40 fps) is not optimal regarding the single beat analysis. This is the reason why we observed a discrepancy in the baseline-to-peak upstroke time of the Ca^2+^ transient, whereas the amplitude is constant and therefore receptive to isoproterenol. Cameras with higher quantum efficiency and FPS will certainly provide new insights in the Ca^2+^ transient analysis and in the cell shortening. Nevertheless, other published algorithms demonstrated a similar response to a positive inotropic agent, including accelerated Ca^2+^ decay and time to peak, even though a lower temporal resolution of 25 fps was applied [32].

The analysis of the Ca^2+^ handling and contractile behavior of isolated cardiomyocytes is a valuable tool for the study of cardiovascular pathophysiology. Remarkably, more information will be available for analyzing dose-dependent effects of inotropic drugs (i.e., isoproterenol) on the aforementioned parameters. This allows greater understanding of how the cardiomyocyte contraction is affected by different diseases, drugs, or genetic manipulations. Our method is an easy and useful tool that generates relevant data regarding the cardiomyocyte functionality. We expect that this tool might help to expand the study of functional features of cardiomyocytes without the need for expensive equipment and software or vast programming expertise.

## Data Availability

The data that support the findings of this study are available from the corresponding author (F.B.), upon reasonable request.

## Supplementary Materials

The following supporting information can be downloaded at: https://www.mdpi.com/article/10.3390/biomedicines10030640/s1. Figure S1: Linear regression correlating Isoproterenol-treated (Iso) cardiomyocytes with untreated cells; Figure S2: Linear regression correlating Isoproterenol-treated (Iso) cardiomyocytes.
