# Supplementary material for: Cell Shortening and Calcium Homeostasis Analysis in Adult Cardiomyocytes via a New Software Tool
Source: Biomedicines. 2022 Mar 10;10(3):640. doi: 10.3390/biomedicines10030640 (PMC8945339; doi:10.3390/biomedicines10030640)
Supplement: Supplementary file 1 [file biomedicines-10-00640-s001.zip › biomedicines-1552940-supplementary.pdf]

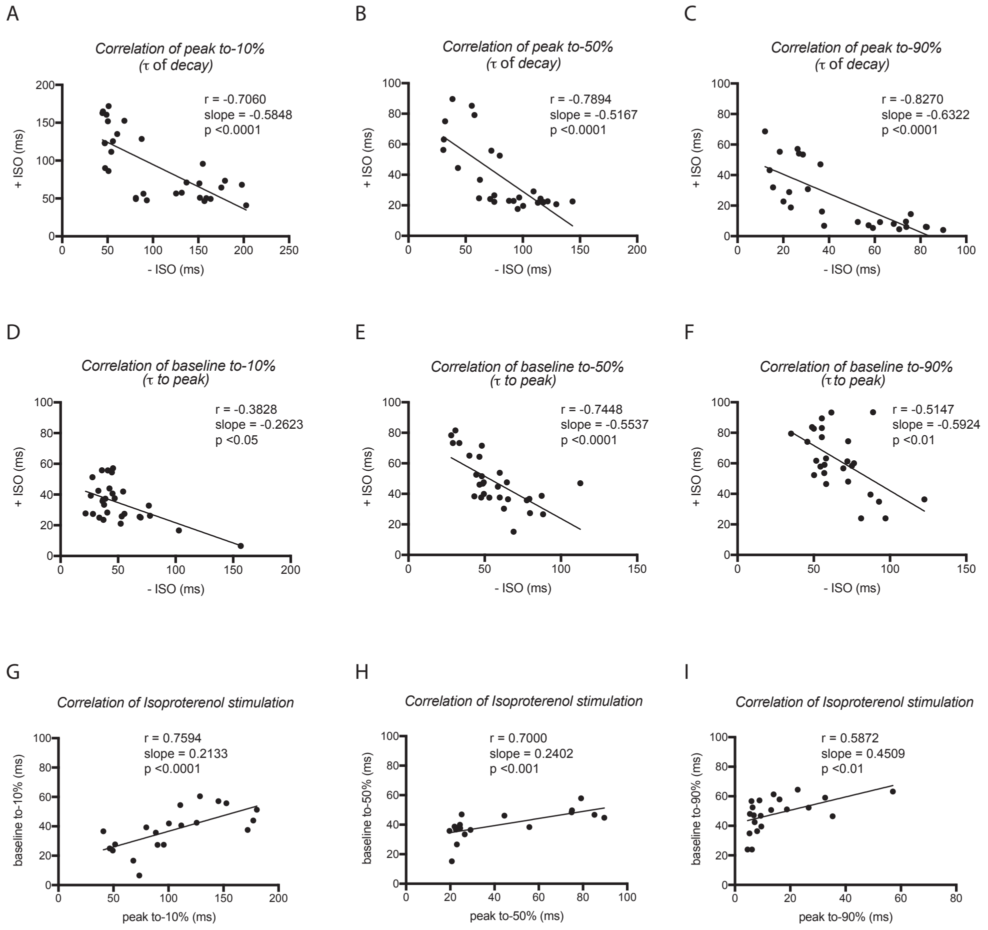

Figure S1. **(A-C)** Linear regression correlating Isoproterenol-treated (Iso) cardiomyocytes with untreated cells. A significant negative correlation was present in τ of decay, in the time for the transient to return to the 10%, to the 50% and to the 90% of the peak during the recovery phase of the transient. **(D-F)** Linear regression correlating Isoproterenol-treated (Iso) cardiomyocytes. Negative correlation between untreated and Iso-treated cells analysis of τ to peak, regarding the time for the transient to reach 10%, 50% and 90% during the rising phase. **(G-I)** Positive correlation in Iso-treated cardiomyocytes between baseline to-10% and peak to-10%, between baseline to-50% and peak to-50% and between baseline to-50% and peak to-50%. The relationships between each pair of variables was evaluated by Spearman correlation coefficient (confidence interval: 95%). p-value of <0.05 was considered significant.

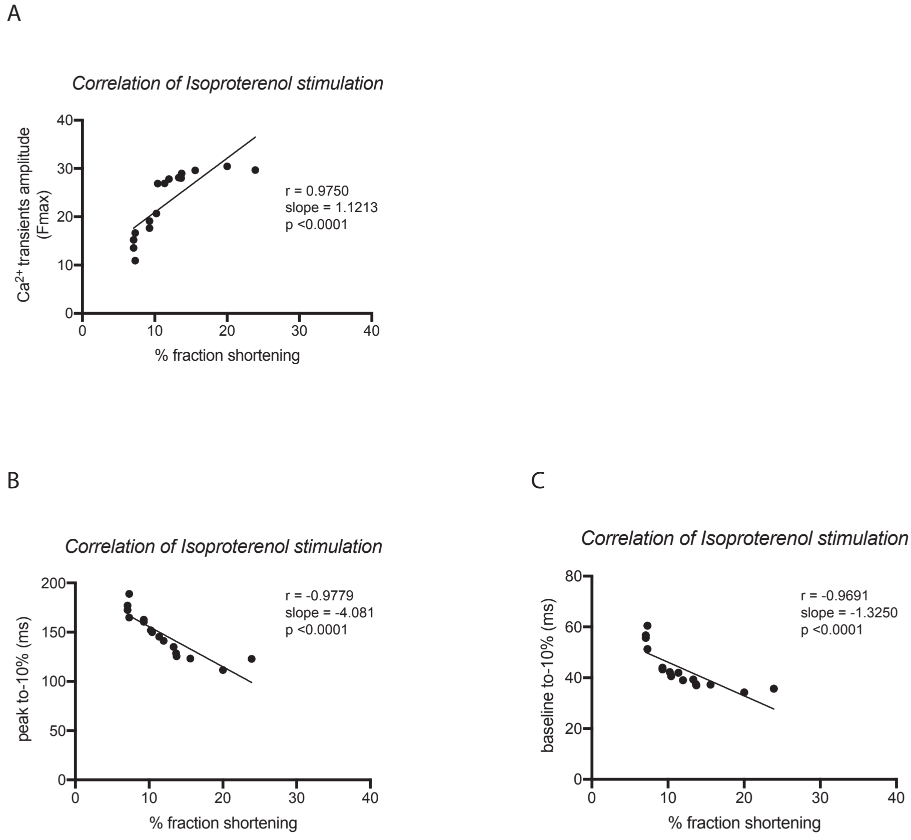

Figure S2. **(A)** Linear regression correlating Ca^{2+}transients amplitude (Fmax) and % fraction shortening in Isoproterenol-treated cardiomyocytes. **(B)** Linear regression correlating % fraction shortening and τ of decay (time for the transient to return to the 10% ; peak to-10%) during the recovery phase of the transient. **(C)** Linear regression correlating % fraction shortening and τ to peak (time for the transient to reach 10% ; baseline to-10%) during the rising phase. The relationships between each pair of variables was evaluated by Spearman correlation coefficient (confidence interval: 95%). p-value of <0.05 was considered significant.
